# Supplementary material for: The interactome of intact mitochondria by cross-linking mass spectrometry provides evidence for coexisting respiratory supercomplexes
Source: Mol Cell Proteomics. 2017 Dec 8;17(2):216–32. doi: 10.1074/mcp.RA117.000470 (PMC5795388; doi:10.1074/mcp.RA117.000470)
Supplement: Supplemental Data [file supp_RA117.000470_133922_0_supp_23978_fzffwf.zip › spectra_annotation/mito_spectra_annotation/76-1-7-1-7-1.pdf]

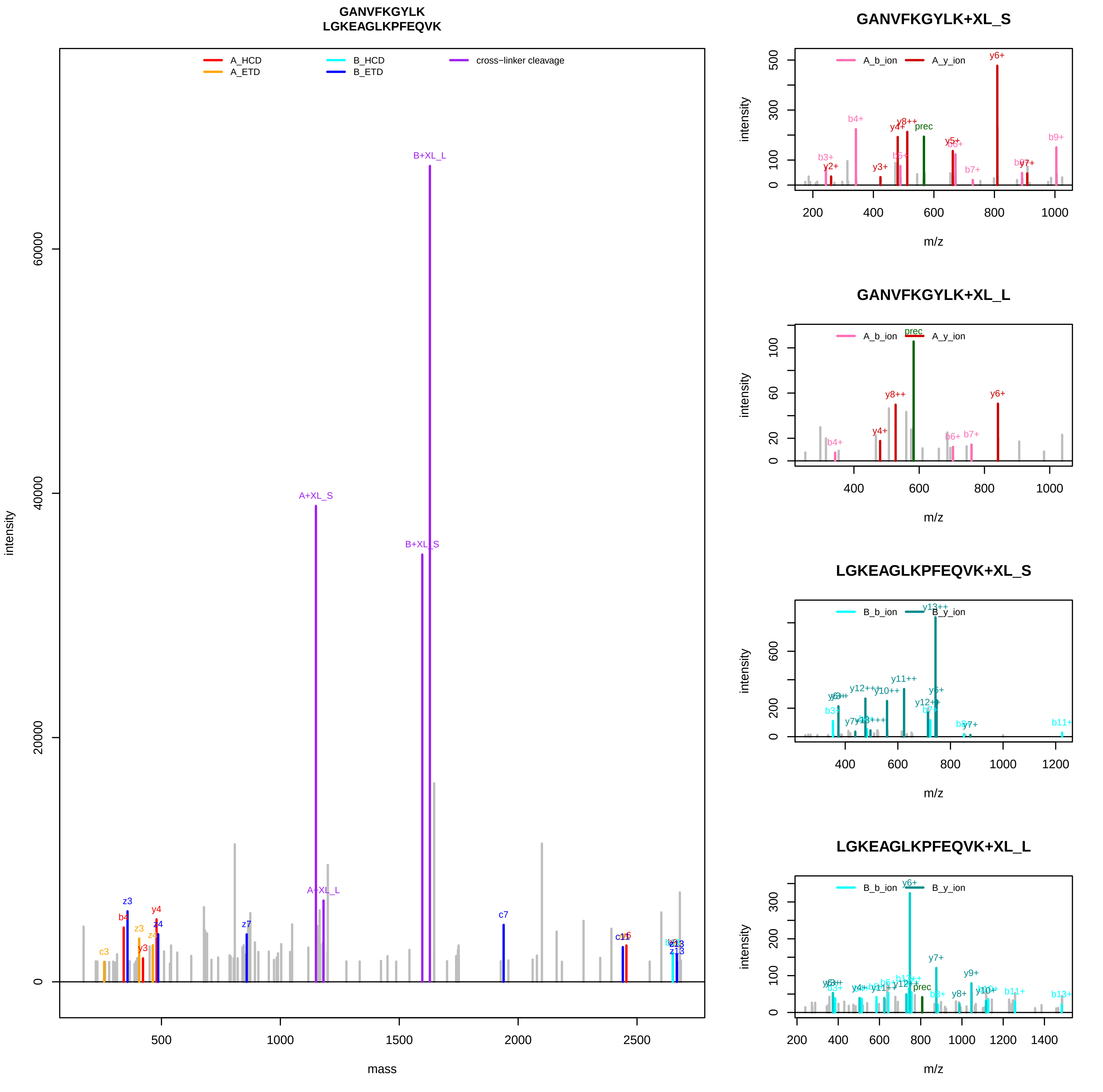
GANVFKGYLK
LGKEAGLKPFEQVK
A_HCD
A_ETD
B_HCD
B_ETD
cross−linker cleavage
B+XL_L
A+XL_S
B+XL_S
A+XL_L
c3
b4
z3
z3
y3
y4
z4
z4
z7
c7
c11
z13
intensity
mass
0
20000
40000
60000
500
1000
1500
2000
2500
GANVFKGYLK+XL_S
A_b_ion
A_y_ion
y6+
b4+
y8++
y4+
prec
b3+
y2+
y3+
b5+
y5+
b6+
b7+
b9+
intensity
m/z
GANVFKGYLK+XL_L
A_b_ion
prec
A_y_ion
y8++
y4+
b4+
b6+
b7+
y6+
LGKEAGLKPFEQVK+XL_S
B_b_ion
y13++
B_y_ion
y11++
y12++
y10++
y6+
b3+
y7+
b8+
b11+
LGKEAGLKPFEQVK+XL_L
B_b_ion
y6+
B_y_ion
y7+
y9+
b3+
y4+
y11++
y12++
prec
b8+
y8+
y10+
b11+
b13+
